# Disease burden and antimicrobial resistance of invasive group B streptococcus among infants in China: a protocol for a national prospective observational study

**DOI:** 10.1186/s12879-017-2475-9

**Published:** 2017-05-31

**Authors:** Wenjing Ji, Haiying Liu, Zhengjiang Jin, Aimin Wang, Xiaoping Mu, Xiaosong Qin, Weidong Wang, Chunyan Gao, Yuning Zhu, Xiaodan Feng, Jine Lei, Shangyang She, Lan Jiang, Jing Liu, Shuhua Yang, Zeshi Liu, Gang Li, Qiuhong Li, Dawen Guo, Muhammad Majid Aziz, Ali Hassan Gillani, Yu Fang

**Affiliations:** 1 The Department of Pharmacy Administration and Clinical Pharmacy, School of Pharmacy, Xi’an Jiaotong University, 76 Yanta West Road, Xi’an, Shaanxi 710061 People’s Republic of China; 2 The Center for Drug Safety and Policy Research, Xi’an Jiaotong University, Xi’an, Shaanxi China; 3 The Global Health Institute, Xi’an Jiaotong University, Xi’an, Shaanxi China; 4 Shaanxi Center for Health Reform and Development Research, Xi’an, Shaanxi China; 5 Clinical Laboratory, Guangzhou Women and Children’s Medical Center, Guangzhou Medical University, Guangzhou, Guangdong China; 6 Department of Clinical Laboratory, Hubei Maternal and Child Health Hospital, Wuhan, Hubei China; 7 Clinical Microbiology Laboratory, Children’s Hospital of Fudan University, Shanghai, China; 8 Clinical Laboratory, Guangdong Women and Children’s Hospital, Guangzhou Medical University, Guangzhou, Guangdong China; 9 Clinical Laboratory, Shengjing Hospital, China Medical University, Shenyang, Liaoning China; 10 Department of Science and Education, Changsha Hospital for Maternal and Child Health, Changsha, Hunan China; 11 Clinical Laboratory, Tangshan Maternal and Child Health Care Hospital, Tangshan, Hebei China; 12 Key Laboratory of Reproductive Genetics (Zhejiang University), Ministry of Education, Clinical Laboratory, Women’s Hospital, Zhejiang University, Zhejiang, Hangzhou China; 13 Clinical Laboratory, Nanjing Maternity and Child Health Care Hospital, Nanjing, Jiangsu China; 14 Clinical Laboratory, The First Affiliated Hospital of Xi’an Jiaotong University, Xi’an, Shaanxi China; 15 Clinical Laboratory, The Maternal and Child Health Hospital of Guangxi Zhuang Autonomous Region, Nanning, Guangxi China; 16 Clinical laboratory, Maternal and Child Health Care Hospital of Uygur Autonomous Region, Urumqi, Xinjiang, China; 17 Clinical Laboratory, Beijing Obstetrics and Gynecology Hospital, Capital Medical University, Beijing, China; 18 Department of Laboratory Medicine, Tianjin Central Hospital of Gynecology Obstetrics, Tianjin, China; 19 Clinical Laboratory, The Second Affiliated Hospital of Xi’an Jiaotong University, Xi’an, Shaanxi China; 20 Medical Experimental Center, General Hospital of Ningxia Medical University, Yinchuan, Ningxia China; 21 Clinical Laboratory, Chongqing Health Center for Women and Children, Chongqing, China; 22 Department of Microbiology, The First Affiliated Hospital, Harbin Medical University, Harbin, Helongjiang China

**Keywords:** Group B streptococcus, Incidence, Case fatality ratio, Serotype, Genotype, Antimicrobial resistance, Resistance gene

## Abstract

**Background:**

Group B Streptococcus (GBS) is a cause of neonatal sepsis, pneumonia, and meningitis that can lead to neurological sequelae in infants less than 3 months of age. The GBS disease burden is not known in China, therefore it cannot receive major attention. The main objectives of this study are the evaluation of the incidence of neonatal GBS infection, GBS case-fatality ratio, its serotypes and genotypes, bacterial resistance, clinical treatment and outcomes in China.

**Methods:**

We are conducting a nation-wide, population-based, multi-center, prospective, observational cohort study in China from May 2016 to December 2017. Eighteen large urban tertiary care hospitals from 16 provinces were selected that cover the eastern, southern, western, northern and central regions of China. Meanwhile, we retrospectively collected data and GBS strains from January 2015 to April 2016 from selected hospitals. The incidence rate per 1000 live births will be defined as the total number of confirmed GBS cases born in the selected hospitals divided by the number of live births in the hospitals during the study period. All GBS cases detected in selected hospitals will be used to calculate the case-fatality ratio and for the typing analysis. GBS isolates will be serotyped using the Strep-B-Latex® rapid latex agglutination test for serotyping of Group B streptococci. Multi-locus sequence typing (MLST) will be performed by sequencing the internal fragments of seven house-keeping genes. Antimicrobial susceptibility will be tested per interpretive standards established by the Clinical and Laboratory Standards Institute. The presence of the common resistance genes *ermA, ermB, mefA, tetI, tetO and tetM* will be tested by PCR.

**Discussion:**

We are conducting the first national study to estimate the invasive GBS disease burden and antimicrobial resistance of GBS among infants in China. Study findings will provide important evidence for improving clinical practice to ensure timely diagnosis of GBS disease and decisions for preventive measures. Surveillance of antimicrobial resistance will promote the rational use of antimicrobials.

**Trial registration:**

The study was retrospectively registered at http://clinicaltrials.gov on June 13, 2016. It was granted a registration number of “NCT02812576”.

## Background

Group B Streptococcus (GBS) is a cause of many neonatal infectious diseases including sepsis, pneumonia and meningitis. Although antimicrobial prophylaxis has significantly reduced the incidence of GBS infection, the mortality rate remains high [1–3]. Furthermore, it is estimated that approximately half of GBS cases have moderate or severe neurological consequences [4, 5]. Infant invasive GBS disease is usually divided into two phases based on age at presentation. Early onset disease (EOD) occurs within 0–6 days of birth, late onset disease (LOD) occurs within 7–90 days of birth. The predominant risk factor for EOD is colonization by GBS of the maternal gastro-intestinal or genitourinary tracts [6].

There is currently no GBS vaccine available. Thus, prevention of prenatal GBS remains a massive challenge. The Centers for Disease Control and Prevention (CDC) in the United States (US) recommended guidelines for Intrapartum Antibiotic Prophylaxis (IAP) in 1996 [7], and the preventive measures of IAP are based on either culture screening (prenatal colonization of GBS) or a risk-based strategy (colonization with GBS, gestational age < 37 completed weeks, and longer duration of membrane rupture, among others). These guidelines were updated in 2002 [8] and 2010 [9], and have recommended universal culture-based screening of all pregnant women at 35–37 weeks of gestation since 2002, reducing the incidence of EOD in the US and other high-income countries [10–12].

Currently, there are no standard guidelines for GBS screening and prevention in China. To our knowledge, few hospitals perform IAP following a risk-based strategy, however, almost no hospital strictly follows the US CDC guidelines. Moreover, no program exists to monitor the prevalence of GBS infection. Nevertheless, two previous studies in China (Beijing and Shenzhen) revealed that GBS is a leading cause of neurological sequelae and high mortality in infants [13, 14]. Similarly, a previous pilot study conducted in two urban hospitals demonstrated a total incidence (0.28/1000 live births) of EOD and LOD among infants, however, this study suffered from underestimation [15]. In addition, further analysis of GBS strains from the pilot study found a new multi-drug resistant cluster of GBS caused by serotype III in China [16]. However, these studies were not truly representative of a large population of China due to the small sample-size restrained areas.

Hence, there is an urgent need for nationwide surveillance data. This will determine the national disease burden and provide surveillance of antimicrobial resistance. The findings of this study will provide strong evidence to improve clinical practice and will be a milestone in the development of precise, optimal preventive measures. Additionally, data pertaining to antimicrobial resistance in this disease will be helpful for the development of future rational antimicrobial-prescribing guidelines. This nation-wide, population-based epidemiological data of GBS disease will aid the assessment of prospective interventions, such as antimicrobial prophylaxis and future vaccine development nationally or globally. These data will be helpful for the allocation of health funds to curtail disease and future research.

## Methods and analysis

### Study objectives

We have designed a suitable study with excellent research and ethical protocols for a better understanding of the disease burden and antimicrobial resistance of invasive GBS in China. The target population includes infants aged from 0 to 90 days. The possible outcomes are given as follows:

1. Incidence
2. Case-fatality ratio (CFR)
3. Susceptibility to specific antimicrobials
4. Spread of GBS types:
  1. Serotyping
  2. Genotyping
5. Detection of common GBS antimicrobial-resistance genes

### Study design and setting

**Fig. 1:**
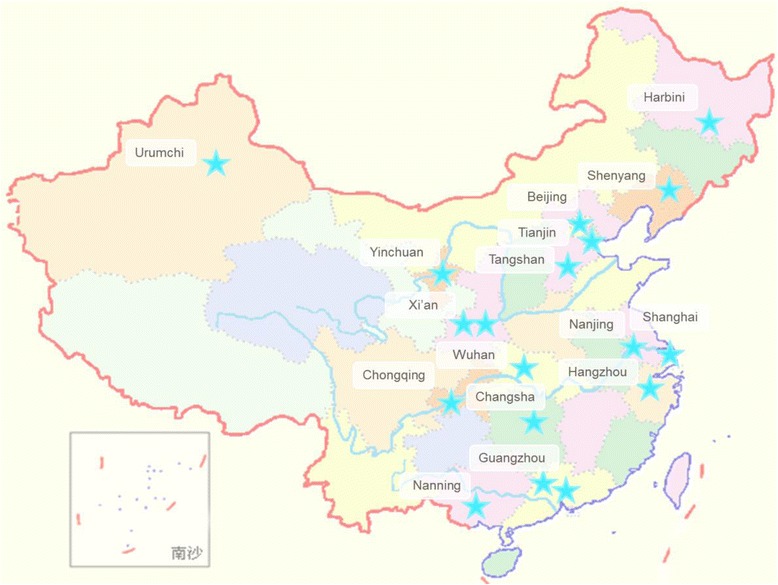
Selected hospital locations throughout China. This figure indicates that the eighteen large urban tertiary hospitals from 16 provinces provide good coverage of the eastern, southern, western, northern and central regions of China. It also demonstrates the study sample has good representativeness

We are conducting a national population-based, multi-center, prospective, observational cohort study in China. As hospitals in China do not have a fixed catchment service area, we chose cities where study hospitals are located near dense populations to ensure maximum coverage. Considering the expected low overall incidence of GBS infection among infants, and the challenge of identifying GBS cases in Chinese primary health care institutions lacking appropriate professionals and laboratory equipment, we set five specific inclusion criteria for site selection: (1) A study hospital must be a large, urban, tertiary hospital; (2) a hospital must have adequate research capabilities and facilities to conduct the study, such as adequate laboratory facilities and competent staff for the identification, processing and storage of GBS isolates; (3) a hospital must have enough time to devote to this study; (4) a hospital must be willing to participate in this research; and (5) according to the geographic regional divisions of China, at least one hospital must come from each region as follows: Northeast China (NE), North China (NC), Northwest China (NW), East China (EC), Central China (CC), South China (SC), and Southwest China (SW). Following a preliminary screening, in which a number of hospitals did not meet at least one of the above criteria, we ultimately selected 18 hospitals from 16 provinces of China to obtain a representative sample. The locations of the 18 hospitals provide greater coverage of the regions of China (Fig. 1).

Among these, 12 hospitals are large maternity and children’s hospitals, providing both obstetric and pediatric care service, and 5 are considered large, complete hospitals. One is exclusively a children’s hospital.

The period of the prospective study runs from May 2016 to December 2017. To complete the retrospective study, data will be collected from clinical charts, hospital and laboratory records specifically from January 2015 to April 2016.

### Sample size

The actual incidence of GBS disease in China is currently unknown. From previous global reports, we have assumed a total incidence (EOD and LOD) of 0.53 cases per 1000 live births [17]. The expected number of GBS cases may therefore be 127 per 240,000 expected live births within the 18 months of the prospective study period.

### Study population

This study may include:

1. Infants born in a selected hospital, which we refer to as “inborn cases”.

2. Infants born in a non-selected hospital who go to a selected hospital for treatment, which we refer to as “out-born cases”.

Inclusion criteria:

(1) Positive culture for GBS from one or more normally sterile sites, such as blood and CSF.

(2) Younger than 90 days old at the time of GBS confirmation.

(3) Written informed consent of parents or legal guardians.

### Study procedures and participant recruitment

**Fig. 2:**
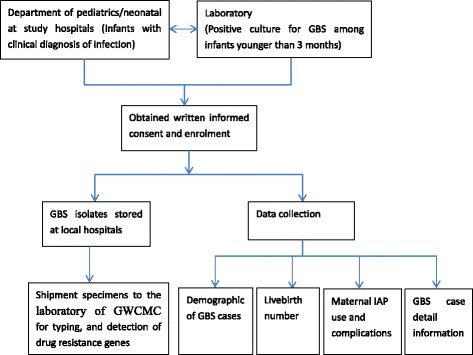
Overview of the study procedures and subject enrollment. This figure describes the study procedures and the enrollment process

All selected hospitals were given a clinical protocol to evaluate and investigate the infected neonates. Identification of GBS cases is based on uniform diagnostic criteria. For any infant presenting with clinical symptoms or signs consistent with suspected GBS cases, including but not limited to fever, poor feeding, breathing problems, heart rate and blood pressure abnormalities, reduced movement, fussiness, bluish-colored skin, seizures, limpness or stiffness, a blood culture is minimally required before antimicrobial administration. Upon laboratory confirmation from at least one of the normally sterile sites, the investigator will contact the parents or guardians of the GBS (+ve) infant for acquisition of consent if the case meets the inclusion criteria. Laboratory personnel are required to monitor the existing electronic lab records system weekly to ensure capture of all GBS cases. All GBS isolates from selected hospitals will be centralized and analyzed at the Joint Commission International certified laboratory of Guangzhou Women and Children’s Medical Center (GWCMC) (Fig. 2).

### GBS culture and identification

Isolation, cultivation and identification of GBS strains will be determined in local laboratories. Sterile samples will be inoculated in culture bottles (BioMerienx, French) or (BD BACTEC™) and incubated with automated blood culture systems. GBS strains will be grown at 37 °C in 5–10% CO_2_ in trypticase soy agar supplemented with 5% sheep’s blood for 18–24 h, according to the manufacturer’s instructions.

### Preparation of frozen stocks of GBS isolates

With a sterile swab, a sweep of colonies (5 cm^2^ or 20–50 colonies) will be collected and the bacteria will be suspended in 1.8 ml cryovials containing 1 ml STGG (Skim milk, Tryptone, Glucose, Glycerol) medium (prepared as per appendix below) by rotating the swab while pressing it against the side of the tube to release the bacteria into the medium. Frozen stocks will be prepared in duplicate, with one to be stored locally as a backup. GBS strains will be cultured in local hospitals and centralized for serotype and genotype analysis in the lab of GWCMC. A laboratory manual will be provided to the laboratories of selected hospitals to provide guidance in the preparation, storage and shipping of samples.

### Serotyping by latex agglutination

GBS strains will be serotyped using the standard Strep-B-Latex® rapid latex agglutination method (Statens Serum Institute, Hillerød, Denmark).

### Multi-locus sequence typing analysis

Polymerase chain reaction (PCR) products, the genomic DNA of GBS, will be purified and bidirectionally sequenced (Beijing Genomics Institute). Multi-locus sequence typing (MLST) will be performed by sequencing the internal fragments of seven house-keeping genes (adhP, pheS, atr, glnA, sdhA, glcK and tkt). MLST will be performed using primers that were described for *S. agalactiae* MLST.

(as detailed at: http://pubmlst.org/sagalactiae/info/primers.shtml).

Alleles and sequence types (STs) of all GBS isolates will be determined by comparing the sequences with those in the *S. agalactiae* MLST.

(as detailed at: http://pubmlst.org/sagalactiae/).

### Antimicrobial susceptibility test

Antimicrobial susceptibility of GBS isolates to 5 antimicrobials (penicillin G, ampicillin, cefazolin, clindamycin, and erythromycin) will be determined using the AST-GP67 (VITEK 2 COMPACT) or manual K-B method at local hospitals, as per interpretive standards established by the Clinical and Laboratory Standards Institute. In addition, detection of resistance genes will be performed in the GWCMC lab. The presence of the common resistance genes *ermA, ermB, mefA, tetI, tetO* and *tetM* will be investigated by PCR. The primer and PCR conditions require the modification of previously described methods [18, 19].

### Data management and statistical analysis

For infants enrolled in this study, data are collected using a paper case report form (CRF) by the investigator, who will sign and date. The CRF collects the variables listed in Table 1.

**Table 1:** List of variables in the Case Report Form

Category	Items	Measures
Infants	Demographic information	Birth hospital, date of birth, gender, birth weight, and gestational age
	Clinical information	Date of onset of first symptoms, child’s admission date, symptoms, and diagnosis
	Laboratory information	Date of sample collection, culture site, culture result, whether patient received antimicrobials prior to sterile sample collection
	Antibacterial therapy	Antimicrobial susceptibility results, antibacterial agent’s name, dose, and duration
	Discharge results	Discharge date, outcome and sequelae
Maternal	Demographic information	Maternal age, mode of delivery
	Clinical information	Risk factors/complications of pregnancy during or after delivery
	Laboratory information	GBS screening and result
	Antibacterial therapy	Antibacterial treatment information

Training regarding completion of these forms will be provided to the investigators. Data from paper CRFs will be entered into a computer database.

Descriptive analysis will be provided for variables including age, gender, birth weight, gestational age, hospitalized duration, antimicrobial usage, and outcome at discharge, among others, categorized by selected hospitals, regions and by disease onset for comparison. The number of GBS cases will be reported overall, by site, onset of disease, and GBS typing. The incidence rate, expressed per 1000 live births, will be computed as the total number of confirmed GBS cases born in the selected hospitals divided by the number of livebirths in the study hospitals during the study period. The case-fatality ratio will be expressed as the percentage (%) of cases of GBS in this study where the outcome recorded at discharge is “died” among GBS cases identified in selected hospitals. For both the incidence rate and case-fatality ratio, 95% confidence intervals (CI) will be calculated using the Wilson interval method.

### Ethical considerations

The protocol, informed consent form (ICF), CRF and other relevant study documents were reviewed and approved by the Xi’an Jiaotong University’s Research Ethics Committee and the Medical Ethical Committees of selected hospitals.

We will obtain voluntary informed written consent from parents or guardians for study inclusion. We will provide the information orally and written in an understandable format regarding the study. Parents / guardians of the subjects have the opportunity to ask about details of the study regarding participation and withdrawal from the study. The investigators will provide a copy of the signed informed consent to the subject and will maintain the original form at the local site.

For each enrolled subject, we will assign a unique study identification number. The CRF and other study documents do not include any identifying information to maintain confidential records of the participants.

### Dissemination

We anticipate that the results of this study will be relevant for health professionals including clinicians, laboratory personnel and relevant preventive policy-makers. Hence, we will disseminate the findings of this study through different channels, such as scientific articles in international peer-reviewed journals and presentations at high-level national and international conferences. Preparation of the study results will be in accordance with the current guidelines of the Strengthening the Reporting of Observational Studies in Epidemiology (STROBE).

(as detailed at: http://www.strobe-statement.org/.)

## Discussion

GBS disease has not been well-recognized or documented in China. This is the first national study to explore the invasive GBS disease burden and antimicrobial resistance among infants in China to address this significant research gap.

First, robust data will provide basic evidence for medical care personnel and the public to improve knowledge of GBS infections.

Second, the improvement of awareness will be helpful for the control of GBS infections, thereby reducing mortality. In addition, this is necessary for the improvement of clinical practices to ensure timely and correct diagnosis of GBS disease.

Third, key information from the surveillance of antimicrobial resistance will support guidelines for rational prescribing attitudes for antimicrobials.

Fourth, the serotyping results will be vital for future GBS vaccine selection in China.

Finally, the findings from the 18 selected hospitals located in different geographical regions will provide information from large hospitals disseminated throughout all Chinese regions; this might facilitate different prevention measures for invasive GBS infections.

There are several limitations to this study. Selection bias is one potential limitation. GBS cases born in non-selected hospitals cannot be included until they seek any sort of therapy at a selected hospital. Disease onset may occur after discharge from the hospital, and once the family has moved out of the catchment areas of study hospitals, it will be difficult to enroll the GBS case. To minimize this risk, the selected hospitals are major tertiary hospitals with good facilities and medical services and are the preferred hospitals when an infant is sick.

Secondary or primary health care institutions and rural hospitals are not included in this study. Therefore, this will lead to the limiting of the representativeness of the findings from this study to urban China.

Several small variations at each study site for blood collection, volume and blood culture systems may have minor effects; therefore, we have provided uniform protocols to each selected hospital, and GBS strains will be centralized in GWCMC for typing analysis.

## Abbreviations

CDC: Centers for Disease Control and Prevention
CFR: case fatality ratio
CI: confidence intervals
CRF: case report form
EOD: early onset disease
GBS: Group B Streptococcus
GWCMC: Guangzhou Women and Children’s Medical Center
IAP: intrapartum antibiotic prophylaxis
ICF: informed consent form
JCI: Joint Commission International
MLST: Multi-locus sequence typing
PCR: polymerase chain reaction
ST: sequence types
STROBE: Strengthening the Reporting of Observational Studies in Epidemiology
